# Pearls collections: What we can learn about infectious disease and cancer

**DOI:** 10.1371/journal.ppat.1006915

**Published:** 2018-03-29

**Authors:** Laura J. Knoll, Deborah A. Hogan, John M. Leong, Joseph Heitman, Richard C. Condit

**Affiliations:** 1 Department of Medical Microbiology and Immunology, University of Wisconsin–Madison, Madison, Wisconsin, United States of America; 2 Department of Microbiology and Immunology, Geisel School of Medicine at Dartmouth, Hanover, New Hampshire, United States of America; 3 Department of Molecular Biology and Microbiology, Tufts University School of Medicine, Boston, Massachusetts, United States of America; 4 Departments of Molecular Genetics and Microbiology (MGM), Pharmacology and Cancer Biology, and Medicine, Duke University Medical Center, Durham, North Carolina, United States of America; 5 Department of Molecular Genetics and Microbiology, University of Florida, Gainesville, Florida, United States of America

“Although infectious agents are major contributors to cancer incidence worldwide, the importance of infectious agents in cancer causation remains relatively underappreciated by the general public and even the scientific community. In addition, mechanisms contributing to infection-related cancers and development of potential prevention and treatment approaches are arguably understudied. The development and implementation of the hepatitis B vaccine (HBV) to reduce the incidence of liver cancer and of human papillomavirus (HPV) vaccines to reduce the incidence of cervical and other HPV-associated cancers in both men and women has increased awareness of the importance and potential avenues for cancer prevention through infectious disease modulation, and more such examples are sure to follow.”

Michael B. Kastan, MD, PhD, Executive Director of the Duke Cancer Institute and William and Jane Shingleton Professor, Department of Pharmacology and Cancer Biology and Department of Pediatrics, Duke University Medical Center

## Introduction

Infections are estimated to be responsible for up to 25% to 50% of all cancers that occur in humans. In light of this significant health burden caused by infectious agents, we commissioned a series of experts to write and contribute reviews on this topic for the *PLOS Pathogens* Pearls review series. A total of 18 Pearls have now been published on this topic, and this editorial overview seeks to highlight this series and contribute to the impact of this collection of essays on this topic by aggregating and collecting thought on the subject. Eight Pearls cover viruses that cause or are linked to cancer, four Pearls cover bacterial pathogens and the microbiome and associations with cancer, and five Pearls cover parasites that have been linked to cancer. One final Pearl is included that covers the exciting and recent area of infections caused by tumor cells, in Tasmanian devils, dogs, and molluscs, including clams. Therefore, the pantheon of five agents that cause infections in humans (viruses, bacteria, fungi, parasites, and prions) are joined now by a sixth infectious modality: infectious tumor cells [1]. The topic of infectious agents in cancer is important in understanding the etiology of cancer, in potential treatments for cancer as highlighted by several Pearls in this series, and in its prevention. There are currently two “cancer” vaccines that are available and in clinical use, and these are for hepatitis B (hepatocellular carcinoma) and human papilloma virus (cervical cancer and others). As we look to the future, we expect that further infectious causes and links to cancer will be discovered and that as cancer therapy and prevention advance, strategies will include treating or preventing infections in the armamentarium. We hope that you find this collection of Pearls essays thought-provoking and stimulating and welcome the submission of future contributions to *PLOS Pathogens* in this important arena.

## Viruses and cancer

Viruses account for an estimated 10% to 15% of human cancers [2]. Currently, the known cancer-causing viruses in humans include seven viruses comprising five virus families [2, 3]. Epstein–Barr virus (EBV)—a herpesvirus—is associated with a variety of malignancies, including for Burkitt lymphoma, diffuse large B cell lymphoma, Hodgkin lymphoma, nasopharyngeal carcinoma, gastric adenocarcinoma, leiomyosarcoma, and posttransplant lymphoproliferative disease. Kaposi sarcoma herpesvirus is the causative agent in Kaposi sarcoma, primary effusion lymphoma, and multicentric Castleman disease. Hepatitis B virus, a hepadnavirus, and hepatitis C virus, a flavivirus, both cause hepatocellular carcinoma. Human T-lymphotropic virus-1, a retrovirus, is responsible for adult T-cell leukemia. Human genital papillomavirus is the causative agent for cervical carcinoma, vulvar cancer, anal cancer, and a number of head and neck cancers. Finally, Merkel cell polyomavirus is responsible for a specific skin cancer called Merkel cell carcinoma. The mechanisms by which viruses cause cancer are under intense investigation and are varied but generally fall into two broad categories: direct and indirect. Direct causation represents the action of viral genes, known in this context as oncogenes, on cellular processes. Indirect causation represents the outcome of chronic inflammation resulting from a persistent viral infection. In no case is cancer the normal outcome of a virus infection. Rather, it is an incidental side effect of the action of viral gene products evolved for the normal process of infection, as in direct causation, or the circumstances of the immune response to infection, as in indirect causation. The identification of viruses as the etiological agent of a number of common cancers provides a unique opportunity to prevent cancer through the use of either vaccination or antiviral drug therapy. Ironically, due to their unique ability to target and kill cells selectively and stimulate a robust immune response, viruses are being developed as “oncolytic” agents to treat cancer.

This compendium of PLOS Pearls includes eight articles that together comprise a survey of the role of viruses in cancer, including mechanism, prevention, and oncolytic agents. Four articles address the mechanism or viral oncogenesis. Moore and Chang explore mechanisms in general and focus on the role of common commensal viruses in cancer [4]. Cavallin et al. discuss the complex mechanisms involved in the induction of Kaposi sarcoma by Kaposi sarcoma herpesvirus [5], and Price and Luftig describe the complexity of EBV latency and its implications for EBV oncogenesis [6]. McBride and Warburton examine the unique role that viral DNA integration into the cellular genome plays in papillomavirus oncogenesis [7]. Three articles address prevention of cancer using antiviral vaccines or drugs. DiMaio provides a succinct history of vaccination, culminating in the development of vaccines for hepatitis B virus and human papillomavirus [8]. Pogoda et al. explore in more depth the development of vaccines for human papillomavirus [9]. Horner and Naggie present an analysis of the successes and challenges surrounding the development of antiviral drugs to treat hepatitis C infections [10]. Lastly, a contribution by Cattaneo and Russell probes the use and potential of viruses to treat cancer, using as an example a remarkable success story on the use of the measles vaccine to treat multiple myeloma [11]. Together, these articles provide insight into the role of viruses in cancer and the hope for cancer therapy that this knowledge brings. The role of viruses in the cause and treatment of cancer is an exciting and expanding area of research, and we can expect significant advances in the future that provide both basic insights into cancer and promise for cancer therapy.

## Bacteria and cancer

The gastric bacterium *Helicobacter pylori* provided the earliest unequivocal link between bacteria and cancer. In the 1970s, gastritis was recognized as a risk factor for the development of stomach cancer, and in the 1980s, Warren and Marshall revolutionized our view of bacteria in cancer by definitively linking peptic ulcer disease to *H*. *pylori* infection. Today, the association between cancer and bacteria is well ensconced in our thinking, but our understanding of the mechanistic bases of this etiological link is still being developed.

Noto et al. [12] explore the potential complexities of this link in “The gastric microbiome, its interaction with *H*. *pylori*, and its potential role in the progression to stomach cancer.” In particular, it is perplexing that while 50% of the population is colonized by *H*. *pylori*, fewer than 3% of *H*. *pylori*–positive individuals develop gastric carcinoma or lymphoma. The authors highlight work from human-based studies as well as in murine models that begin to explain these statistics. Studies have revealed the importance of strain differences, host differences, and the host’s microbiome in dictating the outcome of *H*. *pylori* infection. Because *H*. *pylori* can contribute to changes in the microbiota composition and microbiota may influence the course of disease, the relationship between the pathogen and the microbiota is likely complex and dynamic.

Whitmore and Lamont, in “Oral Bacteria and cancer” [13], describe links between oral bacteria and oral squamous cell carcinoma, one of the most commonly reported cancers. The two genera implicated are *Porphyromonas* and *Fusobacterium*, and cancer has been associated with both the presence of these bacteria or—in the case of *Porphyromonas*—bacterium-specific antibodies. Within these genera, specific species, including *P*. *gingivalis* and *F*. *nucleatum*, modulate intracellular persistence and have immune-disruptive properties [14], and a specific *F*. *nucleatum* factor activates signaling in colon cancer cells through direct interaction with a host receptor [15].

Fulbright et al. (2017) [16] examine the potential roles for bacteria in tumorigenesis utilizing—as a start—a framework set forth by Hanahan and Weinburg [17] that describes the progression of a normal cell in a healthy context to a cancer cell in a complex tumor environment. The steps during this transformation include changes in the regulation of cell proliferation, genome stability, metabolism, immune evasion, and the generation of specific tumor microenvironments. In “The microbiome and the hallmarks of cancer,” Fulbright et al. use the cancer hallmarks framework to describe how either specific microbes or the combined activities of complex microbiota can lead to increased cell proliferation, reduced genome instability, and cancer cell immune evasion. Although most of the data on links between bacteria and cancer are in the context of gastric and colorectal cancers, the authors highlight more recent work exploring the effects of the microbiota on cancer development and progression at more distal intestinal sites.

Finally, Koh [18] discusses the perturbations of the microbiota due to aggressive prophylactic antibiotic therapy and the effects of these perturbations on the treatment of cancer patients and other individuals undergoing hematopoietic stem cell transplants. Risks resulting from a perturbed microbiota include infection and posttransplant graft versus host disease. Interventions that may prevent or mitigate the effects of microbiota perturbation are discussed.

## Parasites and cancer

Similar to other pathogenic microbes, parasites also elicit chronic inflammation that can induce cancer formation. The term parasite in this section refers to the historic definition of parasites, meaning eukaryotic pathogens that are not fungi. This historical definition is different from the common definition: an organism that lives in or on a host and derives benefit at the host's expense. Eukaryotic parasites are divided into two groups: single-cell protozoa and multicellular helminths or worms. Both protozoa and helminths can form long-term infections in humans whose chronic inflammation causes cancer. Two well-known examples of parasitic infections that induce cancer are the flatworms that cause schistosomiasis and the protozoa that cause malaria. Descriptions of *Schistosoma*-induced malignancies date back to the 1950s, and *Plasmodium falciparum*–induced Burkitt lymphoma was first described in 1962 [19]. Burkitt lymphoma arises from germinal center B cells of persons coinfected with EBV and the parasite *P*. *falciparum*. Recent work has shown that *P*. *falciparum* up-regulates activation-induced cytidine deaminase (AID) [20], which changes a cytosine (C):guanine (G) base pair into a uracil (U):G mismatch and induces double-strand breaks. In their Pearl contribution, Thorley-Lawson et al. discuss how, if this induction occurs in EBV-infected germinal centers, induced translocations will more likely be tolerated, leading to Burkitt lymphoma [21].

Another protozoan that has recently been shown to be associated with increased prostate cancer is *Trichomonas vaginalis* [22–24], and this infectious cause of cancer is covered by Sutcliffe et al. in their Pearls contribution [22]. Approximately 170 million people have a chronic, sexually transmitted *T*. *vaginalis* infection. *T*. *vaginalis* is included in the United States Centers for Disease Control and Prevention (CDC) list of the neglected parasitic infections, which are a group of five parasitic diseases that have been targeted by the CDC as priorities for public health action. The mechanism of *T*. *vaginalis*–enhanced prostate cancer is not fully characterized yet, but *T*. *vaginalis* colonizes throughout the prostate [25], and it is thought that the prostate is a reservoir for *T*. *vaginalis* in asymptomatic men [26]. Along with chronic inflammation, when *T*. *vaginalis* adheres to prostate epithelial cells, it triggers a signaling cascade of known proto-oncogenes [22]. While the precise mechanism of *T*. *vaginalis*–induced prostate cancer has not been delineated, it is important to note that there is no sterilizing immunity or memory response to *T*. *vaginalis*, which leads to both its persistent infection and common reinfection. However, this lack of immunity also decreases the hope of a vaccine against this infection and its ensuing cancer.

As covered by Brindley and Loukas in their Pearl contribution [27], several flatworms are associated with increased multiple cancer types. In the life cycle of the blood fluke *S*. *haematobium*, humans are the definitive hosts from which thousands of eggs travel from the parents in the circulation to the bladder, to be released to the environment in the urine. If those eggs become trapped in the bladder wall, the inflammation can cause squamous cell carcinoma of the bladder [28]. The eggs of *S*. *japonicum* and *S*. *mansoni* travel from the circulation to the intestinal tract to be shed in feces, but they can also get caught in the liver during their migration, leading to fibrosis and increased liver cancer, both of which are exacerbated by coinfection with hepatitis viruses [29]. Deposition of *S*. *japonicum* and *S*. *mansoni* eggs in the large intestine can lead to schistosomal granulomatous disease and colorectal cancer. It is less well known that infections with the liver flukes *Opisthorchis viverrini* and *Clonorchis sinensis* can lead to a cancer of the bile duct called cholangiocarcinoma. Mechanisms of carcinogenesis are multifactorial and include both the induction of cancer-causing innate immune responses, such as reactive oxygen species and reactive nitrogen species, as well as parasite metabolites that trigger a chronic inflammatory response. Vaccines that target these metabolic enzymes will likely protect against helminth infections and/or the cancer they induce.

## Infectious agents and cancer treatments

While microbial-induced inflammation can contribute to cancer, science can change the landscape and utilize microbes as part of treatment strategy. The ability of infections to suppress neoplastic growth has been well documented and was originally seen by Dr. William Coley in the 1890s when he noticed that patients had remission of their tumors when they contracted bacterial infections [30]. Infections with the parasite *Toxoplasma gondii* were seen in the 1960s to have dramatic antineoplastic activity [31], and recently attenuated mutants show promise as a new immunotherapeutic agent as covered by Fox et al. in their Pearls contribution to the series [32]. The attenuated *Mycobacterium bovis* strain Bacillus Calmette–Guérin is the only therapy approved for nonmuscle invasive bladder cancer by the Food and Drug Administration [33]. Still, the inflammation induced by infections is usually transient, so efforts have also focused on prompting a long-term cytolytic T lymphocyte response. Because of their ability to survive in antigen-presenting cells, the bacteria *Listeria monocytogenes* and *Salmonella enterica* have been genetically manipulated to deliver tumor antigens to the immune system [34]. Viruses, including an oncolytic herpes virus, have been designed to replicate in tumors and produce granulocyte macrophage colony-stimulating factor (GM-CSF) to enhance the antitumor immune responses [35]. Using these attenuated microbes as adjuvants or as the immunotherapy themselves will undoubtedly play a role in the development of multiple cancer therapies. Cancer can be seen as a disease of the immune system, and immunotherapy’s goal is to eliminate tumors by restoring the immune response. In fact, both viruses and bacteria are now being harnessed for oncolytic therapy against cancer, in which their selective infection of the tumor bed can induce new immune responses to the cancer as well as the pathogen, which is thought to be an important adjunct for immunotherapy as covered in a recent review in *PLOS Pathogens* [36].
